# Induction of *Nanog* in neural progenitor cells for adaptive regeneration of ischemic brain

**DOI:** 10.1038/s12276-022-00880-3

**Published:** 2022-11-14

**Authors:** Gyung-Ah Jung, Jin-A Kim, Hwan-Woo Park, Hyemi Lee, Mi-Sook Chang, Kyung-Ok Cho, Byeong-Wook Song, Hyun-Ju Kim, Yunhee Kim Kwon, Il-Hoan Oh

**Affiliations:** 1 Catholic High-Performance Cell Therapy Center & Department of Medical Life Science, College of Medicine, The Catholic University of Korea, Seoul, Korea; 2 Department of Oral Anatomy, Dental Research Institute & School of Dentistry, Seoul National University, Seoul, Korea; 3 Department of Biomedical and Pharmaceutical Sciences, Kyung Hee University, Seoul, Korea; 4 Department of Pharmacology, Department of Biomedicine & Health Sciences, Catholic Neuroscience Institute, Institute of Aging and Metabolic Diseases, College of Medicine, The Catholic University of Korea, Seoul, Korea; 5 College of Medicine, Institute for Bio-Medical Convergence, Catholic Kwandong University, Gangneung-si, 25601 Korea; 6 Institute for Regenerative Medical Research, StemMeditech Inc., Seoul, Korea; 7 Present Address: Department of Cell Biology, Myunggok Medical Research Institute, Konyang University College of Medicine, Daejeon, Korea

**Keywords:** Stem-cell research, Experimental models of disease

## Abstract

NANOG plays a key role in cellular plasticity and the acquisition of the stem cell state during reprogramming, but its role in the regenerative process remains unclear. Here, we show that the induction of NANOG in neuronal cells is necessary for the physiological initiation of neuronal regeneration in response to ischemic stress. Specifically, we found that NANOG was preferentially expressed in undifferentiated neuronal cells, and forced expression of *Nanog* in neural progenitor cells (NPCs) promoted their self-renewing expansion both in ex-vivo slice cultures and in vitro limiting dilution analysis. Notably, the upstream region of the *Nanog* gene contains sequence motifs for hypoxia-inducible factor-1 alpha (HIF-1α). Therefore, cerebral neurons exposed to hypoxia significantly upregulated NANOG expression selectively in primitive (CD133^+^) cells, but not in mature cells, leading to the expansion of NPCs. Notably, up to 80% of the neuronal expansion induced by hypoxia was attributed to NANOG-expressing neuronal cells, whereas knockdown during hypoxia abolished this expansion and was accompanied by the downregulation of other pluripotency-related genes. Moreover, the number of NANOG-expressing neuronal cells were transiently increased in response to ischemic insult, predominantly in the infarct area of brain regions undergoing neurogenesis, but not in non-neurogenic loci. Together, these findings reveal a functional effect of NANOG-induction for the initiation of adaptive neuronal regeneration among heterogeneous NPC subsets, pointing to cellular plasticity as a potential link between regeneration and reprogramming processes.

## Stroke: Regulatory protein key to neuronal regeneration

The NANOG regulatory protein promotes neuronal regeneration by prompting the self-renewal of progenitor cells following a stroke due to blood vessel blockage (ischemic stroke). NANOG is involved in cellular plasticity and reprogramming, including the maintenance of pluripotent stem cells. Gyung-Ah Jung at the Catholic University of Korea in Seoul, South Korea, and co-workers have demonstrated that NANOG is also critical to the brain’s adaptive response to ischemic stroke. They found that NANOG is preferentially expressed in undifferentiated neuronal cells, and is upregulated under hypoxic (low-oxygen) conditions following a stroke. The activity of *Hif-1*, a master regulator gene for a hypoxic response, boosts expression of the *Nanog* gene resulting in hypoxia-induced proliferation of neuronal progenitors. NANOG induction in neuronal progenitor cells trigger a cascade of changes across the brain, including regeneration of brain tissue after hypoxic injury.

## Introduction

Neurogenesis plays a critical role in brain development and the repair of neuronal tissue. During the embryonic stage, neurogenesis occurs primarily in the ventricular zone in the lateral ventricle of the brain. In contrast, in the adult brain, neurogenesis occurs in the subventricular zone (SVZ) of the lateral ventricle in the forebrain and in the subgranular zone (SGZ) of the dentate gyrus in the hippocampus^1–3^. These specific regions of the brain serve as stem cell niches to maintain the stemness of neural stem cells (NSCs)^4,5^. In turn, NSCs generate new neuronal and glial cells to replenish or expand neuronal cell populations throughout life for the maintenance and reorganization of the nervous system^6,7^.

In addition to homeostatic maintenance, NSCs are also responsible for neurogenesis during functional recovery of injured neuronal tissue^2,8–10^. Therefore, ischemic injury such as cerebral stroke induces neurogenesis primarily in the SVZ and promotes neural progenitor migration from the SVZ to the ischemic boundary region^8,11,12^. Ischemia-induced neurogenesis has also been demonstrated in the adult human brain after stroke, with NSCs contributing to functional recovery after ischemic injury^9,13^.

Notably, an increasing number of studies have revealed extensive heterogeneity in endogenous NSCs that contributes to functional recovery after neuronal injury. For example, in addition to the developmental and spatial differences of NSCs^14^, the heterogeneity of their transcriptomes has been observed through the single-cell analysis of postnatal NSCs in the SVZ region, and these differences correlated with distinct NSC characteristics^15,16^. Moreover, this heterogeneity of NSCs is also associated with differences in receptors and their biological responses to various extrinsic signals^17,18^, implying that specific NSC subtypes might be more readily activated after ischemic injury. Therefore, despite the importance of endogenous neurogenesis for the functional recovery of neuronal tissue, the cellular nature and events underlying neurogenesis remain to be characterized.

Recent studies have shown that cellular plasticity can also contribute to neurogenesis by induction of neuron-specific transcription factors^19,20^. This process involves dedifferentiation of glial cells by induction of SOX2 in astrocytes in the mouse striatum into neuroblasts^21^ as well as changes in chromatin structures towards NSC-like states^22^. Accordingly, these studies raise the possibility that neuronal tissues could also be regenerated through an adaptive reprogramming process in response to injury-mediated stress^23–25^.

NANOG is another transcription factor involved in cellular reprogramming that plays an essential role in the acquisition and maintenance of the pluripotent state of stem cells, with its expression being downregulated after stem cell differentiation^26,27^. An increasing number of studies have indicated the dynamic control of NANOG expression during various cellular processes. For example, NANOG expression is reactivated during the reprogramming of somatic cells into pluripotent stem cells induced by expression of OCT4, SOX2, c-MYC, and KLF4^28,29^. Moreover, NANOG expression was reinitiated in various cancer stem cells, contributing to the stem cell-like state of the cancer cells^30,31^.

However, despite these observations, the physiological significance of NANOG as a functional link between cellular reprogramming and the regenerative process, and as a regulator of the dynamic control of neuronal regeneration in response to physiological stimuli is unclear. In this study, we investigated the reactive expression of NANOG in NPCs for the acquisition of stemness and demonstrated that transient induction of NANOG in neuronal cells represents a key cellular event necessary for the regeneration of neuronal cells in response to ischemic insult. Our study provides new insight into the potential link between reactive regeneration and reprogramming factors for the adaptive regeneration of neuronal tissues.

## Materials and methods

### Animals

Postnatal C57BL/6N mice were purchased from Koatech (Seoul, Korea). Transgenic mice for the *Nanog* reporter (*Nanog*:H2B-GFP, stock number 027563) were obtained from the Jackson Laboratories (Bar Harbor, ME). Another transgenic reporter mouse (*Nanog*-GFP, RBRC02290, RIKEN BRC, Japan) was provided by Dr. Kim, Hyung-Bum (Yonsei University). All animal procedures were approved by the IACUC (Institutional Animal Care and Use Committee) at the College of Medicine, Catholic University of Korea (Approval number: CUMS-2015-0048-04).

### Primary embryonic brain progenitor cell cultures

NPCs were isolated from the forebrain of the C57BL/6N mice on an embryonic day (E) 12.5, or from the cerebral cortex (E16.5) from *Nanog*:H2B-GFP mice^32^. The isolated cells were plated at 1 × 10^6^ cells/100 mm^2^ on Petri dishes and cultured in an N2 medium [DMEM:F12 (1:1) (Invitrogen, Carlsbad, CA) containing N2 supplement (Invitrogen)].

The spheres formed in the cultures were subsequently transferred to and cultured in dishes coated with 1 µg/ml fibronectin (Sigma‒Aldrich, St. Louis, MO). For neuronal cells obtained from the cerebellum of mice on a postnatal day (P) 4, single-cell suspensions were prepared and plated on Petri dishes with B27 medium or on laminin-coated dishes (20 µg/ml).

### Reporter assays

The *Nanog* luciferase reporter gene (–5203*Nanog*-*Luc*) was a gift from Dr. A Suzuki^33^. *Nanog-Luc* (0.2 µg) and pCMV-*β-gal* (0.05 µg) were cotransfected into NPCs from E12.5 using Lipofectamine Plus reagent (Invitrogen). For assays to determine transactivation of the *Nanog* promoter, 0.1 or 0.5 µg p*Hif-1*-PA, a constitutively activated form of *Hif-1α*^34^, was cotransfected into cells. Luciferase activity was measured using the Luciferase Assay System (Promega, Madison, WI) according to the manufacturer’s instructions. pcDNA was used as the control, and transfection efficiency was based on normalized β-galactosidase activity level.

### Nanog knockdown by sh-Nanog lentivirus and limiting dilution assays

The lentiviral vectors pFUIPW-rtTA2S-M2, pFTREW-EGFP-miR30, and pFUGW-EGFP-sh-*Nanog* were gifts from Dr. Jungmook Lyu, Konyang University, Korea^35^. P4 cerebellar NPCs were transduced with combinations of two lentiviral vectors (*s*h-*Nanog* and rtTA2S-M2) and incubated for 4 h. The next day, the medium was replaced with a fresh B27 medium change. Three days after infection, the expression of transduced genes driven by a tet-on system was induced by treating the cells with 500 ng/ml doxycycline for 2 days. Transduced (EGFP^+^) cells were plated in limiting dilution dose on a low attached 96-well plate (Corning, Corning, NY) with B27 medium for 3 days under 5% O_2_ or 21% O_2_*.*

### Hypoxic conditions

To test the effects of hypoxia, sh-*Nanog*-transduced NPCs were exposed to hypoxia (5% O_2_) or normoxia (21% O_2_) for 2 days. For phenotypic analysis of NPCs after knockdown, cells cultured under hypoxic conditions were sorted, purified, and seeded (2 × 10^4^ cells/well) on four-well cell culture slides (SPL, Korea) coated with laminin, incubated for 2 h and then fixed with 4% PFA for immunohistochemical staining.

### Overexpression of Nanog and sphere formation assays

A retroviral vector encoding *Nanog* was cloned into a pMIG (MSCV-IRES-GFP) vector^34^ and transduced into NPCs derived from E12.5 mouse brains. Cells infected with the retroviral vector were cultured for 4 days and treated with 10 µM BrdU (BD Pharmingen, San Diego, SD) for 24 hours. For sphere formation assays, transduced (GFP^+^) cells were sorted by fluorescence-activated cell sorting (FACS) and cultured in an N2 medium in serial limiting doses.

### Organotypic spinal cord slice cultures

NPCs isolated from mouse brains on E12.5 were cultured, transduced with a retroviral vector (pMIG-*Nanog*), and cultured for 4 days. They were then treated with 10 µM BrdU for 24 h before sorting for transduced (GFP^+^) cells. The sort-purified cells were then transplanted onto spinal cord slices according to a method previously described^36^. Seven days after culture in the spinal cord, the slice cultures were fixed with 4% PFA and incubated with anti-BrdU (BD), anti-TUJ1 (Merck, Kenilworth, NJ), anti-NESTIN (Merck), anti-GalC (Merck), and anti-GFAP (Merck) antibodies. Immunostaining was visualized by confocal microscopy (Olympus, Japan).

### Immunoblot analysis

Cell lysate proteins were separated by SDS-polyacrylamide gels and transferred to a membrane. The membrane was incubated with the following primary antibodies: anti-NANOG (Novus Biologicals, Littleton, CO), anti-KLF4 (GeneTex, Irvine, CA), anti-SOX2 and anti-c-MYC (Cell Signaling Biotechnology, Danvers, MA), anti-OCT4 (Cell Signaling Biotechnology), or anti-β-ACTIN (Abcam Ltd., Waltham, MA). Protein bands were detected using enhanced chemiluminescence (ECL) (Invitrogen) and a PXi4 imaging system (Syngene, UK).

### Immunofluorescence staining

For phenotyping of NPCs by immunofluorescent staining, spheres were dissociated into single cells using 0.25% trypsin and fixed with 4% PFA in PBS. The cells were permeabilized with PBS containing 0.1% Triton X-100 and incubated with anti-GFP (Abcam Ltd), anti-NESTIN (Millipore, Billerica, MA), anti-TUJ1 (SIGMA-Aldrich), anti-MAP2 (Abcam Ltd), anti-CD133 (Thermo Fisher Scientific), anti-NANOG, anti-GFAP (Agilent Technologies, Santa Clara, CA), anti-CNPase (Abcam Ltd), or anti-PCNA (Abcam Ltd.) antibodies. The cells were subsequently incubated with Alexa 488-anti-rabbit IgG, Alexa 488 or Alexa 594-anti-mouse (Abcam Ltd.), Cy3-conjugated anti-rabbit IgG (Jackson ImmunoResearch, West Grove, PA), or Alexa 594-conjugated anti-rat IgG (Abcam Ltd.). Images were obtained by confocal microscopy, and the cells were counted manually in a blinded manner.

### Cerebral stroke animal model

Cerebral stroke was induced in male C57BL/6 mice as previously described^37^. Briefly, after anesthesia, the ischemic injury was induced by ligation of the right common carotid artery with a 6–0 silk suture. After 2 h of recovery, mice were exposed to a hypoxic chamber (7.5% oxygen, 92.5% nitrogen) for 40 min. Animals were transcardially perfused with PBS, followed by 4% PFA from 1 to 3 days after the hypoxic insult. The brains were frozen and cut into 30-μm-thick sections for immunofluorescence staining.

### RT‒PCR

Total RNA was isolated using TRIzol, and cDNAs were synthesized according to the reverse transcriptase manufacturer’s instructions (SuperScript II, Invitrogen, Carlsbad, CA). RT‒PCR primers were *mNanog*-F: ATGAAGTGCAAGCGGTGGCAGAAA, *mNanog*-R: CCTGGTGGAGTCACAGAGTAGTTC, *mNanog* (transgene)-F:CTTGAACCTCCTCGTTCG, *mNanog* (transgene)-R: CCTGGTGGAGTCACAGAGTAGTTC, *m18sRNA*-F: CGGCTACCACATCCAAGGAA, and *18 sRNA*-R: GCTGGAATTACCGCGGCT.

### Microarray analysis

Microarray analysis was performed by MACROGEN (Korea). The analytical platform was used with an Affymetrix Mouse Gene 2.0 ST Array (Thermo Fisher Scientific). The data were summarized and normalized via the robust multiaverage (RMA) method with Affymetrix Power Tools (APT). The statistical significance of differential expression data was determined by the LPE test. The false discovery rate (FDR) was controlled by adjusting the *p* value using the Benjamini‒Hochberg algorithm. Gene Ontology (http://geneontology.org) and KEGG (http://kegg.jp) gene enrichment and functional annotation analysis of the significant probe list was performed.

### Statistical analysis

The data were expressed as the mean ± standard error of the mean (SEM). Unpaired Student’s *t*-test was performed to determine the significance of a difference between the two groups. One-way ANOVA or two-way ANOVA followed by the Tukey post hoc test was performed to compare three or four groups.

## Results

### NANOG promotes the self-renewal of NPCs

**Fig. 1:**
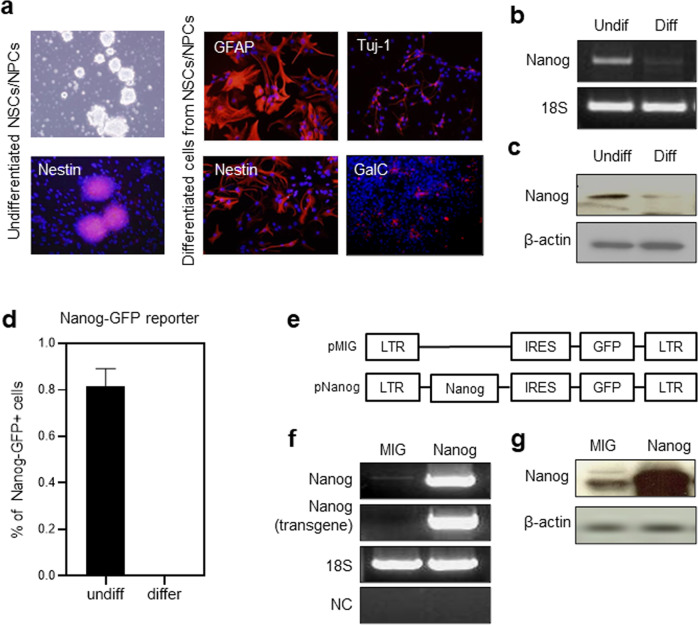
Expression of the NANOG in neuronal cells. **a** Immunostaining of neurospheres before and after differentiation induction. Undifferentiated E12.5 NPCs in sphere form and their differentiated cells were validated by immunostaining for NESTIN (undifferentiated), GFAP (astrocyte), TUJ1 (neuron), and GalC (oligodendrocyte). **b**, **c** Selective expression of NANOG in undifferentiated NPCs. NPCs in neurospheres or differentiated cells were analyzed for expression of *Nanog* in transcript and protein products. Shown are the representative profiles for RT-PCR analysis (**b**) and immunoblotting of the NANOG protein (**c**). **d** Selective expression of NANOG in undifferentiated cells as determined by *Nanog* reporters (*Nanog*-GFP). NPCs obtained from the brains of transgenic mice (*Nanog*-GFP) on E12.5 were similarly induced to differentiate, and the frequency of NANOG-expressing (GFP^+^) cells was determined by flow cytometry (*n* = 6). **e–g** Establishment of NANOG-overexpressing NPCs. A schematic illustration of the retroviral vector (**e**), verification of transgenic expression of *Nanog* in these cells using RT‒PCR (**f**) and immunoblots showing the protein products (**g**) in cells transduced with a control vector (MIG) and vectors encoding *Nanog*. *Nanog* (transgene): PCR products based on primers for the MIG vector; NC negative control.

To investigate the functional role of the *Nanog* gene in neuronal tissue, we first examined its expression in neuronal cells at different stages of differentiation. Thus, undifferentiated neurospheres from the embryonic mouse brain were induced to undergo differentiation, and the NANOG expression levels among the cells were compared (Fig. 1a). As shown in Fig. 1, undifferentiated neurosphere cells expressed higher levels of NANOG than differentiated cells, as determined by the difference in levels of both mRNA transcript and the protein product (Fig. 1b, c). The higher level expression of NANOG in the undifferentiated neurospheres was confirmed by analyzing GFP-expressing neuronal cells derived from transgenic mice, where the expression of green fluorescent protein (GFP) was driven by the *Nanog* promoter (Fig. 1d).

**Fig. 2:**
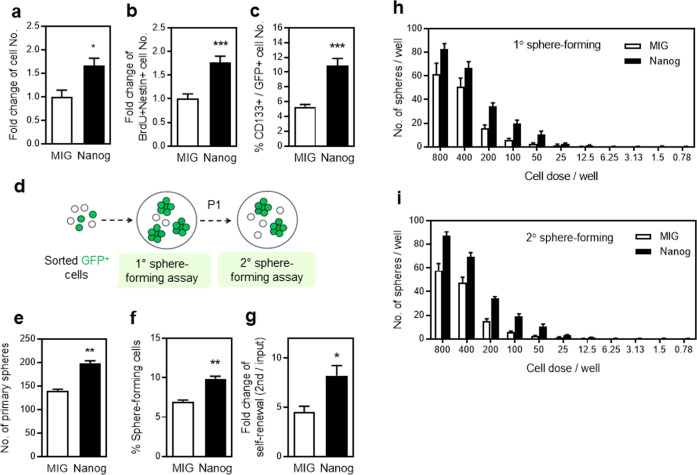
Effects of NANOG overexpression on self-renewal of NPCs. **a**–**c** NPCs transduced with a control or *Nanog*-expressing vector were compared with respect to expansion as measured by total cell number (**a**) (*n* = 3, *p < 0.05), the fold expansion of undifferentiated NPCs determined by numbers of BrdU^+^NESTIN^+^ cells (**b**) (*n* = 6, ***p < 0.001) and frequencies of undifferentiated (CD133^+^) cells among the transduced cell (GFP^+^) population (**c**) (*n* = 5, ***p < 0.001). **d**–**g** Effects of NANOG expression on the self-renewal of neurosphere-forming cells were determined by comparing the frequency of sphere-forming cells in the primary and secondary subcultures of each transduced NPC (GFP^+^). An illustration of the experimental scheme is shown (**d**), and the numbers of primary spheres among the initially seeded plated cells (2 × 10^3^ cells), (**e**) (*n* = 3, **p < 0.01), the percentage (%) of sphere-forming cells (**f**) (*n* = 3, **p < 0.01), and the fold increase in sphere-forming cells during primary and secondary culture (**g**) are shown (*n* = 3, *p < 0.05). **h**, **i** Limiting dilution analysis of neurosphere-forming cells during subculture. NPCs were plated in serial dilutions and the resulting numbers of spheres in each dose were determined in primary culture (**h**) (*n* = 5) and secondary subcultures (**i**) (*n* = 10).

Considering the selective expression of NANOG in undifferentiated neuronal cells, we next forced expression of NANOG in these cells to examine its function. We constructed a retroviral vector expressing *Nanog* along with a GFP-encoding gene and transduced the vector into neurosphere cells to establish NPCs overexpressing NANOG (Fig. 1e–g). NPCs overexpressing NANOG exhibited a significant increase in cell numbers with a significant increase of proliferating NPCs (BrdU^+^NESTIN^+^) and frequency of CD133^+^ cells, the undifferentiated neuronal subsets enriched with sphere-forming cells^38^, compared to control-transduced cells (Fig. 2a–c). Thus, forced expression of NANOG in NPCs promotes the expansion of undifferentiated neuronal subsets.

To further characterize the expansion of NPCs induced by NANOG overexpression, we analyzed the self-renewal of NPCs by performing a serial sphere-forming activity of the sort-purified transduced cells (GFP^+^) (Fig. 2d). We found that NANOG-expressing cells exhibited a higher frequency of sphere-forming cells and underwent higher rates of self-renewal during culture, as determined by frequencies in the secondary neurosphere-forming assay (Fig. 2e–g). Similarly, limiting dilution analysis for sphere-forming cells revealed higher self-renewal of these cells over primary and secondary sphere-forming cells (Fig. 2h, i). These results together indicate that forced expression of NANOG in NPCs promotes their self-renewal for expansion of undifferentiated NPCs during culture.

**Fig. 3:**
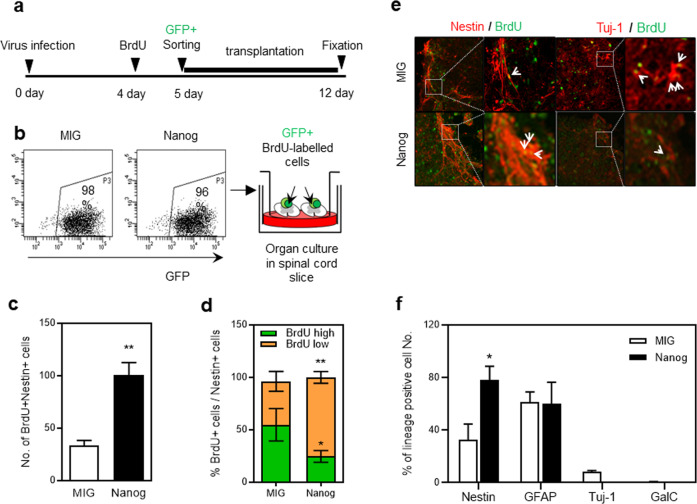
Effects of NANOG expression on NPCs under organotypic spinal cord slice culture conditions. Effects of NANOG expression on NPCs were examined in a spinal cord slice culture model that mimics the in vivo microenvironment. **a** Schematic illustration showing the experiment. After transduction with viral vectors, cells were pulse-labeled with BrdU and sort-purified for transplantation of transduced (GFP^+^) cells in organotypic spinal cord slices for 7 days. **b** Representative flow cytometry profiles for purification of transduced neuronal cells and their transplantation into organotypic spinal cord slices. **c** Numbers of BrdU^+^NESTIN^+^ neuronal cells were compared between MIG- and *Nanog*-transduced cells after 7 days of culture (*n* = 3, **p < 0.01). **d** NESTIN^+^ cells were examined for relative % of highly proliferating (low BrdU intensity) and slow proliferating (high BrdU intensity) cells by immunostaining with an antibody against NESTIN and BrdU (*n* = 3, *p < 0.05, **p < 0.01). **e**, **f** Differentiation pattern of NANOG-expressing NPCs in organotypic spinal cord slice cultures. The transplanted cells were double stained for each lineage marker and BrdU. Representative profiles showing NESTIN and TUJ1 and the relative distribution of differentiated cells of each lineage are shown (**f**) (*n* = 3, *p < 0.05).

Based on the effects of NANOG on NPCs under in vitro culture conditions, we sought to examine the effects under conditions that closely resemble the in vivo microenvironment of neuronal tissues. For this, we employed an organotypic spinal cord slice culture system where cells are transplanted into live slices of spinal cord tissue^36^ to track cell fates in the condition that can more closely resemble the in vivo microenvironment for neuronal cells. Thus, NPCs were transduced with retroviral vectors encoding *Nanog* and pulse-labeled with BrdU, then sort-purified transduced cells (GFP^+^) were transplanted into the organotypic spinal cord slice culture system (Fig. 3a, b). During the 7 days of cultivation on spinal cord slice, the numbers of BrdU^+^NESTIN^+^ cells significantly increased for NANOG-expressing cells than for control-transduced cells (Fig. 3c). In particular, NANOG-expressing cells exhibited significantly higher proportions of BrdU low (highly proliferating) cells among the BrdU^+^NESTIN^+^ cells than the control group (Fig. 3d). Consistent with these findings, lineage analysis of the cultured cells showed a significant increase in the frequency of NESTIN^+^ cells, but not in the frequency of more differentiated (GFAP^+^, TUJ1^+^ or GalC^+^) cells compared to control-transduced cells (Fig. 3e, f).

Together, these results show that upregulation of NANOG in neuronal cells selectively enhances the maintenance of the undifferentiated state by promoting self-renewal both in vitro and in vivo, mimicking microenvironment conditions.

### NANOG promotes the expansion of NPCs in response to hypoxic stimuli

**Fig. 4:**
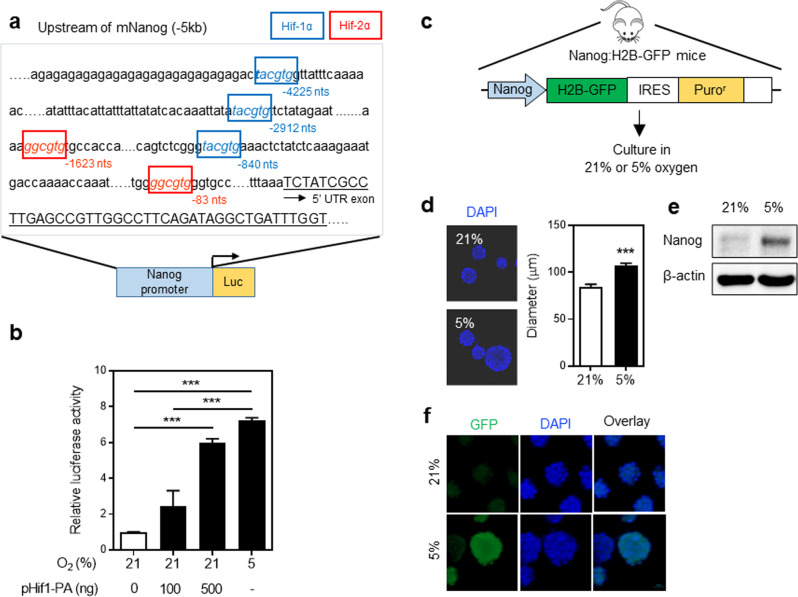
Hypoxia induces NANOG production in neuronal cells. **a** Structure of the *Nanog* promoter/upstream region. Shown is the upstream sequence of the *Nanog* gene and the consensus sequence for HIF-1α (blue box) and HIF-2α (red box) binding, along with the structure of the *Nanog* reporter expressing the luciferase gene. **b** Transactivation of the *Nanog* promoter by hypoxia. E12.5 NPCs were transfected with the *Nanog*-luciferase reporter and exposed to normoxia (21% O_2_) or hypoxia (5% O_2_) or cotransfected with the indicated amounts of constitutively activated *Hif-1α* (p*Hif-1*-PA). The luciferase activity was normalized on the basis of the β-galactosidase activity level. The relative luciferase activities are shown (*n* = 4, ***p < 0.001). **c** Transgenic mice carrying the *Nanog* gene reporter. Schematic showing the structure of the transgenic reporter gene with GFP expression driven by the *Nanog* promoter. **d**–**f** Effects of hypoxia on the transgenic expression of *Nanog* in transgenic mice. **d** The effects of hypoxia on neurosphere size. A representative picture (left panel) and the mean ± SEM showing the diameter of 115 neurospheres (right panel) (***p < 0.001). **e**, **f** Induction of *Nanog* expression by hypoxia in neuronal cells was analyzed by immunoblot analysis using an antibody against NANOG (**e**) and immunofluorescent staining for GFP in neurospheres (**f**).

Given the role of NANOG in the self-renewing expansion of NPCs, we investigated its role in the physiological stimulation of neuronal regeneration. For this, we first examined the effect of hypoxia, which is a physiological condition that triggers the self-renewing proliferation of undifferentiated neuronal cells^39,40^ (Supplementary Fig. 1a–e). Interestingly, in the upstream regions of the *Nanog* gene, we found several consensus binding sites for HIF-1α and HIF-2α, the two master regulatory factors of hypoxic responses^41,42^ (Fig. 4a). This discovery prompted us to ask whether hypoxic stress could induce transactivation of the *Nanog* promoter by HIF-1α. The transactivation assays using this reporter showed that the *Nanog* gene is significantly induced by hypoxia as well as by constitutively activated HIF-1α (p*Hif1*-PA)^34^. These results indicate that hypoxia indeed upregulated *Nanog* gene expression (Fig. 4b).

To confirm the physiological regulation of *Nanog* by hypoxia, we next examined the induction of *Nanog* expression in neuronal tissues exposed to hypoxia. To this end, we employed transgenic mice in which the *Nanog* gene was knocked in with fusion genes encoding a histone and GFP (*H2B*-*GFP*) (Fig. 4c), and neuronal cells from the cerebral cortex were examined for induction of *Nanog* by hypoxic stimuli. Exposure of these neuronal cells to hypoxia increased sphere size compared to normoxia (Fig. 4d) and profoundly increased NANOG in these cells, as determined by immunoblot for NANOG and immunohistochemical staining for GFP (Fig. 4e, f). These results indicate that hypoxia induces upregulation of NANOG in neuronal cells.

**Fig. 5:**
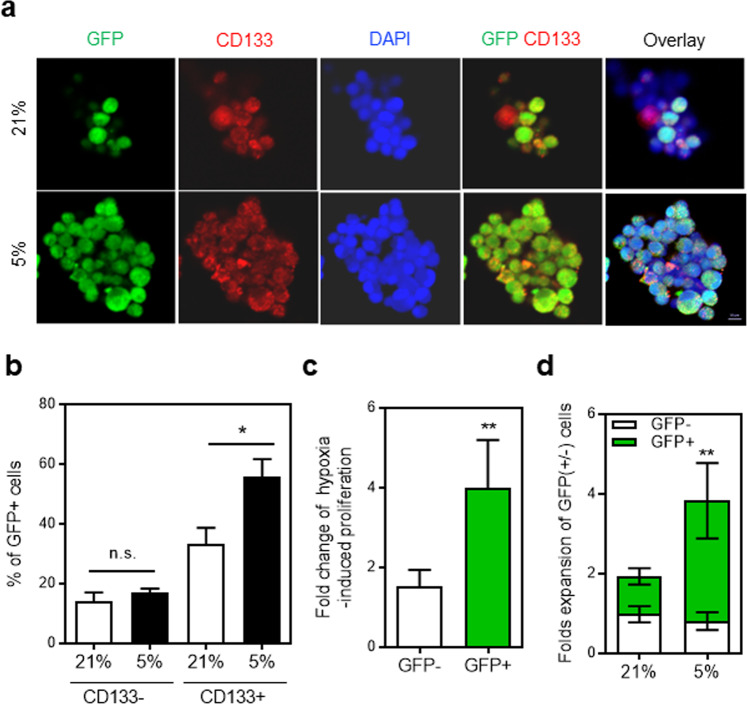
Contribution of NANOG-expressing cells to a hypoxia-induced expansion of NPCs. NPCs from transgenic mice (*Nanog*:*H2B*-*GFP*) were exposed to normoxia or hypoxia and expansion of NANOG-expressing (GFP^+^) and non-expressing (GFP^−^) cells were analyzed along with expression of CD133. **a**, **b** Increase of NANOG-expressing cells in response to hypoxia among differentiated (CD133^−^) and undifferentiated (CD133^+^) neuronal cells. Representative images showing immunofluorescent staining (**a**) and the percentage (%) of NANOG-expressing (GFP^+^) cells after expansion for 3 days (**b**) (*n* = 4, *p < 0.05). **c** Comparison between the hypoxia-induced expansion of NANOG-expressing and non-expressing cells (*n* = 3, **p < 0.01). **d** Contribution of GFP (+) and GFP (−) cells to the hypoxia-induced expansion of NPCs. Three days after culture under normoxic or hypoxic conditions, the increase in cell number compared to the input cell numbers was measured along with the relative percentage (%) of GFP (+) in the expanded cells. Shown are the expansion folds of NPCs under each condition relative to the input cell numbers with % of GFP (+) cells in each expanded cell population marked in green (*n* = 4, **p < 0.01).

Next, we investigated the role of *Nanog*-induction for hypoxia-induced expansion by comparing NANOG expressing (GFP^+^) and non-expressing (GFP^-^) cells, taking into account the heterogeneity of NSCs for distinct biological responses^15–17,43^. We found that undifferentiated (CD133^+^) cells exhibited a significant increase in NANOG induction (GFP^+^), whereas differentiated (CD133^-^) cells did not induce NANOG (Fig. 5a, b). These findings indicate that the induction of NANOG in response to hypoxia occurs selectively in the undifferentiated neuronal cell population. Interestingly, when NANOG (+) and NANOG (−) cells were compared for hypoxia-induced proliferation, NANOG expressing (GFP^+^) cells exhibited a significantly greater increase of neuronal cells (Fig. 5c). Accordingly, for the hypoxia-induced increase of neuronal cells, a predominant (up to 80%) proportion of the cells were NANOG-expressing (GFP^+^) cells, whereas NANOG non-expressing (GFP^-^) cells contributed to the expansion only marginally (Fig. 5d). These findings together indicate that hypoxia-induced expansion of neuronal cells is mostly contributed to by NANOG-expressing NPCs and that these NANOG-expressing NPCs represent a major population in hypoxia-induced neuronal regeneration.

### Physiological significance of NANOG induction for neuronal regeneration

**Fig. 6:**
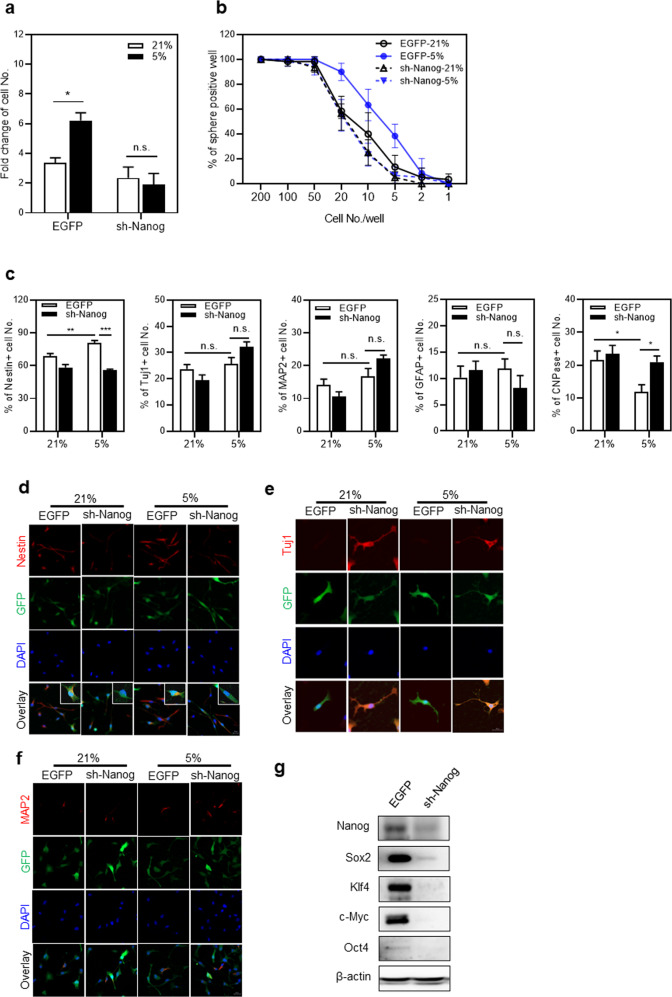
Effects of *Nanog* knockdown on hypoxia-induced expansion and differentiation of NPCs. **a** Influence of *Nanog* KD on the hypoxia-induced expansion of NPCs. The fold expansion of control and sh-*Nanog*-expressing cells in response to hypoxic stimuli is shown (*n* = 6, *p < 0.05). **b** A limiting dilution assay with *Nanog*-KD NPCs. Transduced (EGFP^+^) cells were seeded at 1–200 cells/well and cultured for 3 days under 5% O_2_ or 21% O_2_ conditions. The percentage of the sphere-positive well was calculated by the number of well that existed in the sphere over 50 µm (*n* = 5). **c**–**f** Lineage analysis of NPCs transduced with sh-*Nanog*. After culture under normoxic or hypoxic conditions, transduced (EGFP^+^) cells were sorted and stained for the indicated lineage markers. Shown are the % numbers of cells positively stained for each indicated marker under normoxic and hypoxic conditions (*n* = 10–19 for each lineage, *p < 0.05, **p < 0.01, ***p < 0.001) (**c**) and representative immunostaining images showing NESTIN, MAP2, and TUJ1 (**e**, **f**). **g** Concomitant downregulation of pluripotency genes with KD of *Nanog*. The immunoblot analysis for each indicated pluripotency gene in control and sh-*Nanog*-transduced neuronal cells under hypoxic culture conditions is shown.

To further investigate these findings, we examined the effect of *Nanog* knockdown (KD) on the hypoxia-mediated expansion of neuronal cells. To this end, we transfected neuronal cells with vectors encoding sh-*Nanog* and compared their expansion under hypoxia or normoxia. The hypoxia-induced expansion of neuronal cells was completely abolished in the group expressing sh-*Nanog*, whereas the control group exhibited significant expansion (Fig. 6a). Similarly, the frequencies of neurospheres were significantly increased by hypoxia in the control group, but these increases in neurosphere frequencies were abolished by the expression of sh-*Nanog* (Fig. 6b). These results show that NANOG-induction in NPCs is an essential step for the hypoxia-induced expansion of neuronal cells as well as their maintenance of the undifferentiated state. Importantly, the inhibition of neuronal expansion by sh-*Nanog* under hypoxia was most prominent in the NESTIN (+) cells, while the frequencies of differentiated (TUJ1^+^, MAP2^+,^ GFAP^+^, or CNPase^+^) cells among the transduced cell population were not decreased or increased by *Nanog* KD (Fig. 6c–f). Furthermore, the coordination of reprogramming factors was observed in the hypoxic response of neuronal cells as the knockdown of *Nanog* concomitantly suppressed the expression of other reprogramming factors such as SOX2, KLF4, and c-MYC (Fig. 6g). These results together suggest that induction of NANOG in NPCs might be a key initiation step for hypoxia-induced neuronal regeneration by coordinating reprogramming factors and the regeneration process.

**Fig. 7:**
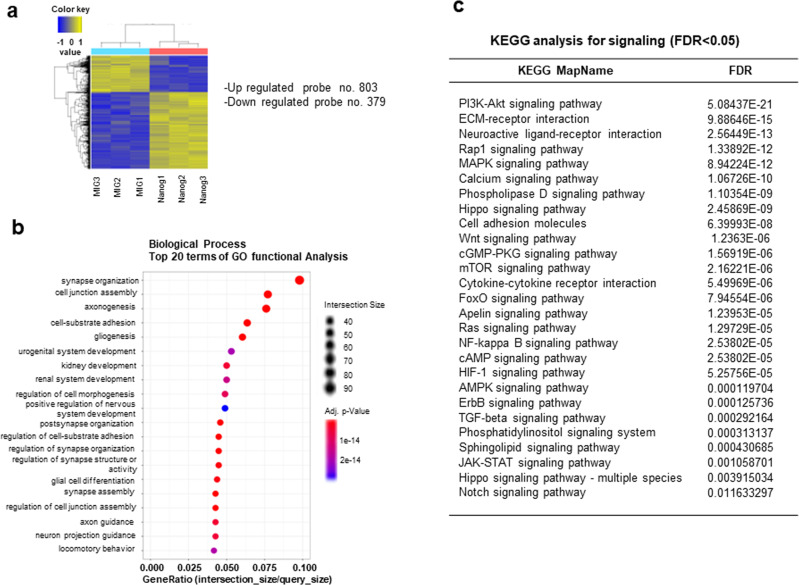
Transcriptome changes induced by overexpression of *Nanog* in NPCs. NPCs obtained from the cerebellum of postnatal day 4 mice were transduced with *Nanog* using a retroviral vector (MIG). Five days after transduction, RNA was purified and subjected to transcriptome analysis by RNA-seq and subjected to by Gene Ontology and KEGG analysis. **a** Heatmap showing two-way hierarchical clustering analysis (Euclidean method, complete linkage). Differentially expressed genes (DEGs) were classified by lpe.*p* < 0.05 and fold change >1.5 between three batches of control (MIG 1–3) and *Nanog*-transduced cells (Nanog 1–3) (*n* = 3). **b** Results of the 20 most enriched terms in the GO functional biological process analysis. Each enriched GO term is shown with adjusted *p* values and the intersection size (gene ratio). **c** KEGG pathway analysis of DEGs between MIG- and *Nanog*-transduced cells. Significantly (FDR <0.05) enriched signaling pathways are shown.

To further explore the molecular changes induced by *Nanog* expression in neuronal cells, we examined the transcriptome changes in NPCs overexpressing *Nanog*. As shown, 803 and 379 genes were significantly (fold change>1.5, *p* < 0.05) up- and downregulated, respectively (Fig. 7a). A Gene Ontology analysis of these differentially expressed genes (DEGs) showed significant enrichment with genes involved in neurogenesis, including synapse organization, axonogenesis, gliogenesis, and nervous system development (adjusted *p* < 1e-14) (Fig. 7b). When analyzed for signaling pathways by KEGG analysis, multiple spectrums of signaling pathways involved in cellular regeneration and proliferation (FDR <0.05) (Fig. 7c). These results together show that NANOG induction in neuronal cells triggers an extensive transcriptomic change that are enriched with genes for neurogenic activities.

**Fig. 8:**
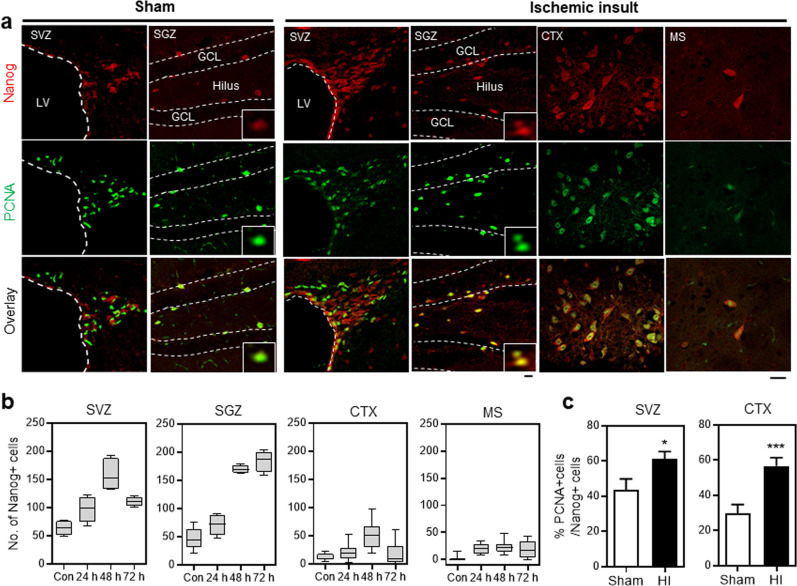
Transient induction of NANOG in brain regions undergoing neurogenesis in response to ischemic insult. Ischemic insult in mice was induced by ligation of the right common carotid artery and exposure to a hypoxic chamber. The spatial distribution of NANOG-expressing cells in mouse brains near the infarct area was examined along with sham-operated mice 24, 48, and 72 h after ischemic insult**. a** Images of immunofluorescence staining of the brain 48 h after hypoxic insult (HI) or sham operation. High-magnitude images of NANOG-expressing cells are displayed in inlets. **b** The numbers of NANOG-expressing cells were counted in each indicated region on day 24, 48 and 72 h after ischemic insult. The numbers of NANOG (−) cells, with upper and lower margins of the boxes representing 75 and 25% of the value, with horizontal bars representing the mean values (*n* = 8 and 4 for control and HI, respectively, for SVZ and SGZ regions, *n* = 8 and 16 for control and HI, respectively, for CTX and MS regions) are shown. Scale bar = 20 μm. **c** Proliferative activity of NANOG-expressing neuronal cells. The brain regions were costained for PCNA and NANOG in an analysis of the proliferation of NANOG-expressing cells. The percentage (%) of proliferating (PCNA^+^) cells among the NANOG-expressing cell population in mouse brains exposed to ischemic insult compared to the corresponding brain regions in sham-operated mice (*n* = 12 for SVZ, *n* = 11 for CTX, *p < 0.05, ***p < 0.001). Scale bar = 20 μm. SVZ subventricular zone, SGZ subgranular zone, CTX cerebral cortex, MS medial septum.

Next, to determine the physiological relevance of the findings with respect to the neurogenic effects of NANOG, we explored whether neuronal levels of NANOG increase in response to a physiological stimulus that triggers regeneration induced by an ischemic insult in the brain. To this end, we analyzed the spatial distribution of NANOG-expressing cells in brains exposed to ischemic insult along with their in vivo proliferative activities. The numbers of NANOG-expressing neuronal cells were predominantly increased around the infarct area in the subventricular zone (SVZ) of lateral ventricles and subgranular zone (SGZ) of the dentate gyrus in the hippocampus within 2–3 days of ischemic insult, but only marginal increases were observed in the cerebral cortex and medial septum (Fig. 8a, b and Supplementary Fig. 2). These results indicate that the brain regions in which hypoxia-induced an increase in NANOG-expressing neuronal cells markedly coincide with specific loci of the brain that exhibited active neurogenesis^1–3,44,45^. However, for both regions of neurons in the SVZ or the cerebral cortex, NANOG-expressing cells exhibited significantly increased staining for proliferating cell nuclear antigen (PCNA)^46^ (Fig. 8c) after hypoxic injury. This finding indicates that NANOG-expressing cells underwent reactive proliferation in response to an ischemic insult to initiate neuronal expansion in multiple areas of neurogenesis in the brain.

Together, these results show that NANOG induction in neuronal tissues is associated with neuronal proliferation in response to ischemic insult, suggesting a role for adaptive neuronal regeneration in response to physiological stress.

## Discussion

Endogenous neurogenesis is a key step in the regeneration of injured neuronal tissues, and is contributed to by heterogenous populations of NSCs^1,2^. Accordingly, understanding the molecular events underlying these regenerative processes and the NSC subsets critical for specific regenerative signals is crucial for understanding neuronal diseases and developing new treatments.

In this study, we show that *Nanog*, the pluripotency gene that is reactivated during cell reprogramming^26,47^, plays another key role in neuronal regeneration in response to hypoxic injury. We first found that NANOG is selectively expressed in the undifferentiated neuronal population and its upregulation promotes their self-renewal and maintenance of undifferentiated NPCs. The role of NANOG in NSCs was also observed in a similar model, where NANOG was induced during self-renewal of NSCs stimulated by Hedgehog signaling in the postnatal cerebellum or medulloblastoma^48^. In addition, NANOG expression was correlated with multipotency or proliferative invasion in a glioblastoma model^49,50^, similarly supporting a role for NANOG in the stemness of normal and malignant neuronal cells.

Interestingly, we found that HIF-1, a master regulator in the hypoxic response, can directly transactivate the *Nanog* promoter, revealing *Nanog* as a downstream target gene. Thus, we demonstrated that neuronal cells exposed to ischemic stress increased NANOG expression and underwent a selective expansion of undifferentiated NPCs. The physiological significance of NANOG induction for the hypoxia-induced proliferation of NPCs was evident from the finding that knockdown of NANOG during hypoxia abolished the expansion of NPCs, and that 80% of the neuronal expansion induced by hypoxia was comprised of the NANOG-expressing cells. Furthermore, the ischemic brain regions exhibiting higher frequencies of NANOG-expressing cells coincided with the brain regions undergoing active neurogenesis, such as the SVZ and SGZ, further supporting the physiological significance of NANOG induction in ischemia-induced neuronal regeneration. However, it is noteworthy that NANOG-expressing cells in areas of less active neurogenesis, such as the cerebral cortex, also exhibited increased proliferating activities in response to ischemic insult. This finding suggests that NANOG induction during neuronal regeneration may be a common event during neurogenesis regardless of the brain area undergoing neuronal regeneration.

Notably, our findings provide new insight into the physiological significance of NANOG induction in the context of NSC heterogeneity. Previous studies have shown extensive heterogeneities of NSCs with respect to spatial or developmental stages^15,16^ as well as to the expression of receptors and their responses to various biological signals^17,18,43^. Hence, it has been postulated that these subsets of NSCs exhibit functional compartmentalization for specialized responses to their microenvironments. However, in our study, *Nanog* KD led to complete abrogation of hypoxia-induced expansion and neurosphere formation in the NPCs, the heterogeneous subpopulations, indicating that induction of NANOG is essential for hypoxia-induced regeneration of neuronal cells beyond their heterogeneity of the subpopulations. Supporting this possibility, the predominant portion of the hypoxia-expanded neuronal cells comprised NANOG-expressing cells, while the expansion of cells that did not express *Nanog* was marginal. Taking these findings together, it is plausible that the induction of NANOG may be a conserved process in the initiation of neuronal regeneration among heterogeneous NSCs. However, further evidence obtained via a cell-trafficking analysis is needed to show the regenerative role played by NANOG in individual NANOG-expressing cells. Similarly, considering that ischemic stroke leads to oxygen deprivation to different degrees in various regions of the brain, and that the hypoxic response is triggered at O_2_ concentrations less than 7% (PaO_2_ <50 mmHg)^51^, potential variations in the hypoxic response and NANOG induction in different brain regions should be investigated in future studies.

Notably, our study highlights another issue: specifically, cellular reprogramming and the regenerative process are connected. Taking the function of NANOG as a key molecule for cellular reprogramming towards pluripotency, our findings suggest that cellular plasticity for reprogramming is at least partially involved in the neuronal regeneration process. Consistent with this possibility, a previous study showed that another reprogramming factor, SOX2, caused dedifferentiation of mature glial cells, astrocytes, and oligodendrocytes into proliferative NPCs, which led to the generation of mature neuron cells^50^.

Notably, we found that the induction of NANOG was coordinated with the induction of other molecules; i.e., while hypoxic stimuli induced NANOG expression in neuronal cells, knockdown of NANOG induced concomitant downregulation of other reprogramming factors (SOX2, c-MYC, and KLF4) (Supplementary Fig. 3). This finding suggests potential coordination of these reprogramming factors in response to environmental cues. In addition, it is also noteworthy that induction of NANOG in ischemic neuronal cells was transient, lasting for 2–3 days after the ischemic insult. Accordingly, one of the differences between cellular reprogramming and the regeneration process may be related to differences in the duration of the expression of reprogramming factor genes. Supporting this possibility, a series of studies have shown that regeneration of injured or aged tissues is facilitated by the transient expression of reprogramming factor genes in response to injury signals^52–54^, which is accompanied by dedifferentiation during the regenerative process^55–59^. Further studies are warranted to elucidate the functional coordination of reprogramming factors in regeneration after tissue injuries with respect to various microenvironments.

In summary, our study shows that NANOG induction is a key molecular event initiating the self-renewing expansion of NPCs in response to hypoxic stimuli and demonstrates its predominant role in the physiological regeneration of neuronal tissues injured by ischemic insult. Our study also provides insights into the functional coordination of reprogramming factors activated by microenvironmental injury signals during the regenerative process.

## Supplementary information

Supplementary Figures
