# Supplementary material for: Induction of Nanog in neural progenitor cells for adaptive regeneration of ischemic brain
Source: Exp Mol Med. 2022 Nov 14;54(11):1955–66. doi: 10.1038/s12276-022-00880-3 (PMC9722910; doi:10.1038/s12276-022-00880-3)
Supplement: Supplementary file 1 — Supplementary Figures [file 12276_2022_880_MOESM1_ESM.pdf]

Supplementary Figures

Supplementary Fig. 1

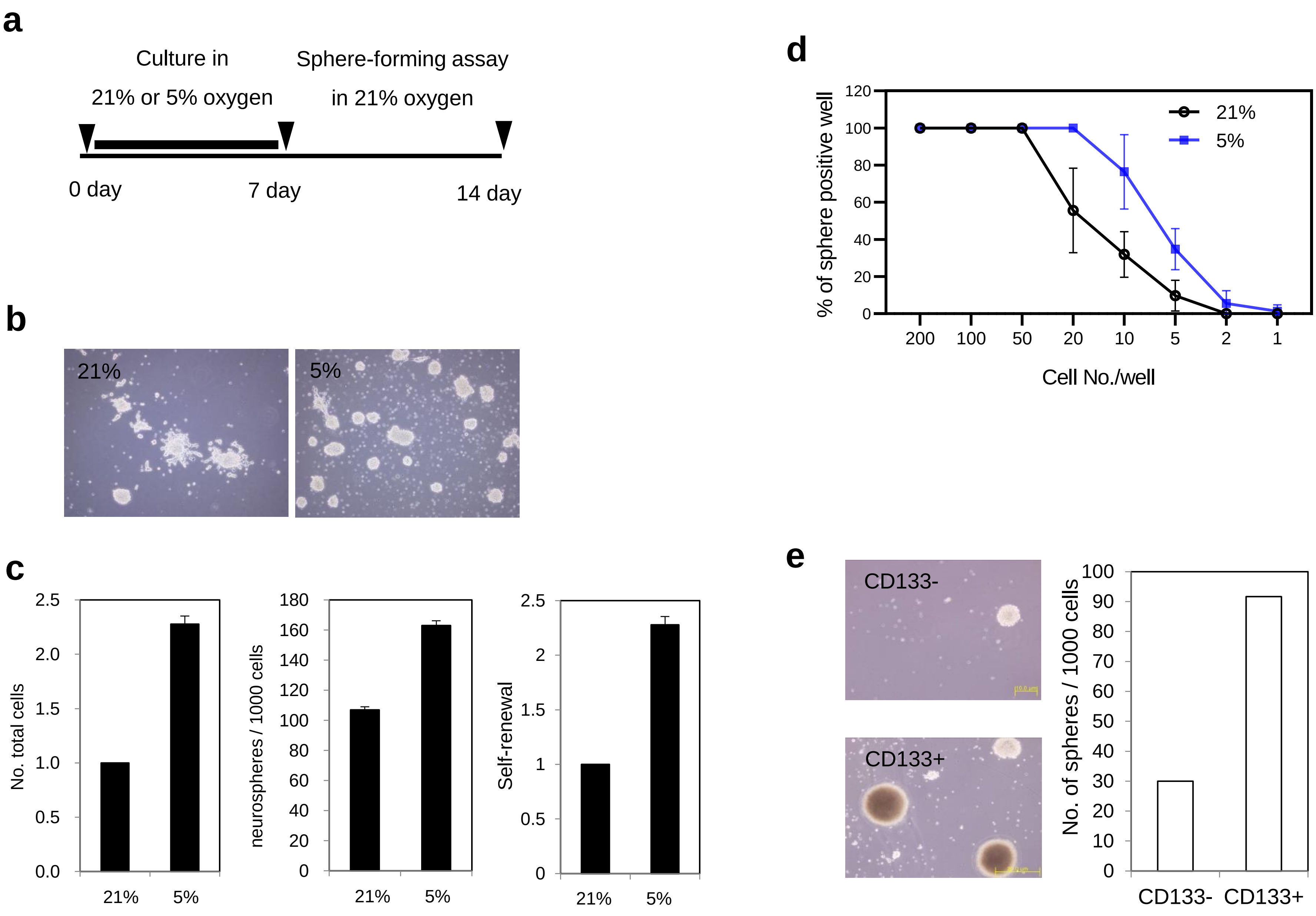

Supplementary Fig. 1. The effects of hypoxia on sphere forming cells and expansion of undifferentiated cells. (a-c) NPCs from E12.4 embryonic mice were plated for sphere formation under normoxia (21% O2) or hypoxia (5% O2) conditions. Thus-formed spheres were visualized by light microscopy and quantified. Shown are the experimental scheme (a) and representative images of the spheres under each condition (b) and numbers of neurospheres during primary and secondary subculture along with self-renewal of neurosphres (c).

(d) Limiting dilution analysis for frequencies of neurosphere-forming cells during hypoxia and normoxia. NPCs were plated in limiting dilution dose for 3 days under normoxia or hypoxia. The resulting numbers of wells positive for neurospheres were counted. Shown are the % of sphere forming cells at each indicated cell numbers plated in the well (n=5).

(e) Correlation of the undifferentiated state and sphere-forming activity of NPCs. NPCs were sort purified into CD133(+) and CD133(-) cells and the sphere forming activity of each population was examined. Shown are the representative light microscope images for each population demonstrating differences in sphere formation (left panel) and frequencies of sphere-forming cells obtained after plating CD133(-) and CD133(+) cells (1000 cells/plate)

Supplementary Fig. 2

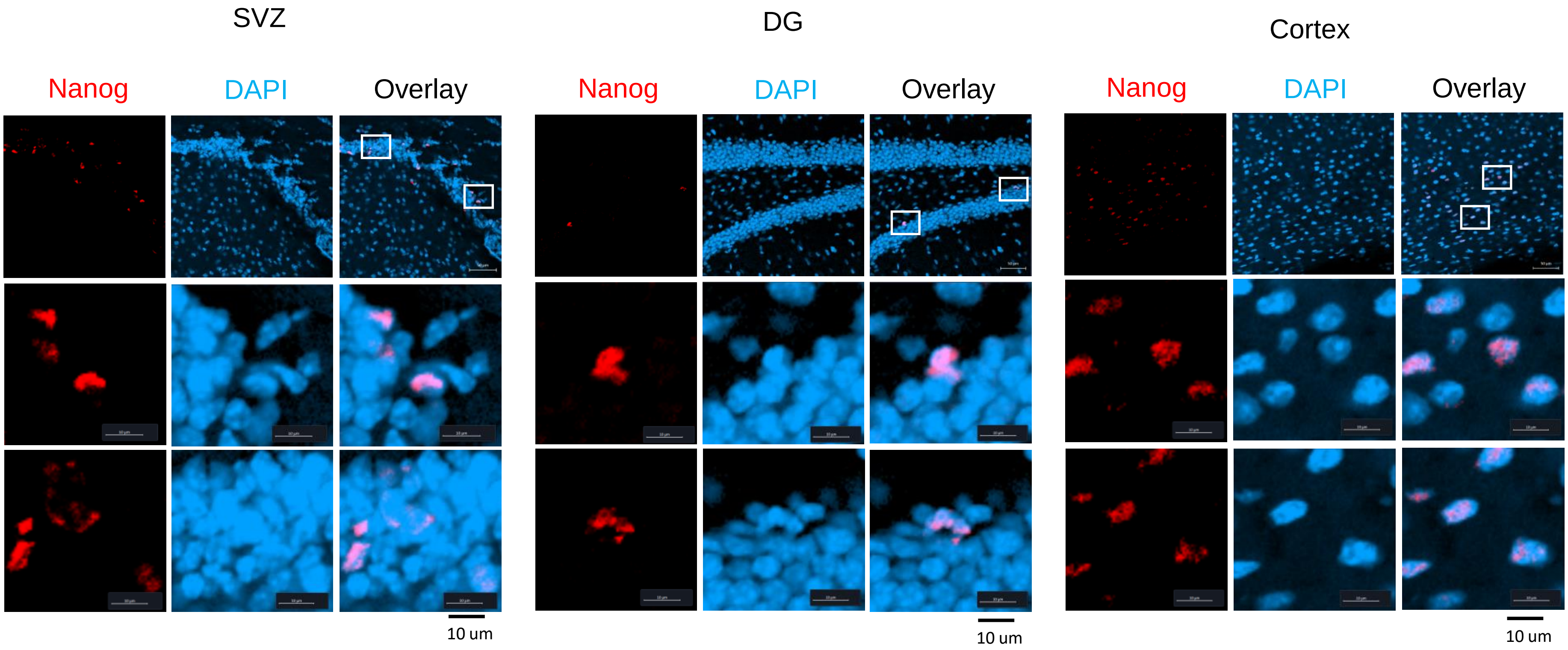

Supplementary Fig. 2. Subcellular localization of nanog in ischemic brain cells.

Ischemic insult in mice was induced by ligation of the right common carotid artery and exposure to a hypoxic chamber. The subcellular distribution of nanog-expressing cells in mice brains near the infarct area were examined by immunofluorescent staining for nanog in SVZ, Dentate gyrus (DG) and cortex region. Shown are the representative images for nanog (red) with DAPI (green) with lower magnification (upper row) and magnification (mid, lower row).

Supplementary Fig. 3

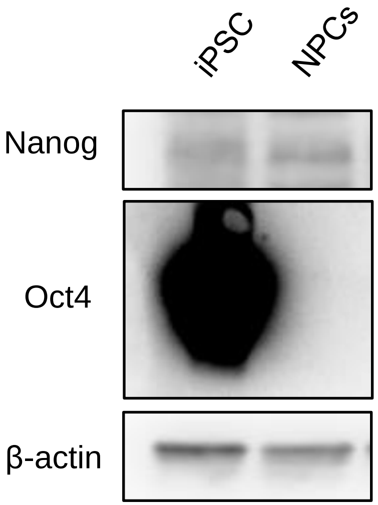

Supplementary Fig. 3. Expression levels of Nanog in NPCs and iPSCs.

Protein expression levels of Nanog in NPCs were visualized by immunoblot along with expression of Oct-4 as a positive marker for iPSCs
